# A comprehensive trait dataset for Terrestrial Arthropods of the Azores: insights for conservation, island ecology and species invasion

**DOI:** 10.3897/BDJ.14.e173221

**Published:** 2026-01-12

**Authors:** Guilherme Oyarzabal, François Rigal, Pedro Cardoso, Isabel R Amorim, Ricardo Costa, Sébastien Lhoumeau, Sophie Wallon, Nuria Macías-Hernández, Sofia Terzopoulou, Kostas A. Triantis, Paulo A. V. Borges

**Affiliations:** 1 University of Azores, CE3C—Centre for Ecology, Evolution and Environmental Changes, Azorean Biodiversity Group, CHANGE —Global Change and Sustainability Institute, School of Agricultural and Environmental Sciences, Rua Capitão João d’Ávila, Pico da Urze, 9700-042, Angra do Heroísmo, Azores, Portugal University of Azores, CE3C—Centre for Ecology, Evolution and Environmental Changes, Azorean Biodiversity Group, CHANGE —Global Change and Sustainability Institute, School of Agricultural and Environmental Sciences, Rua Capitão João d’Ávila, Pico da Urze, 9700-042 Angra do Heroísmo, Azores Portugal; 2 Environment and Microbiology Team, Université de Pau et des Pays de l’Amour, Pau, France Environment and Microbiology Team, Université de Pau et des Pays de l’Amour Pau France; 3 LIBRe – Laboratory for Integrative Biodiversity Research, Finnish Museum of Natural History, University of Helsinki, Helsinki, Finland LIBRe – Laboratory for Integrative Biodiversity Research, Finnish Museum of Natural History, University of Helsinki Helsinki Finland; 4 CE3C - Centre for Ecology, Evolution and Environmental Changes, CHANGE – Global Change and Sustainability Institute, Faculty of Sciences, University of Lisbon, Lisbon, Portugal CE3C - Centre for Ecology, Evolution and Environmental Changes, CHANGE – Global Change and Sustainability Institute, Faculty of Sciences, University of Lisbon Lisbon Portugal; 5 University of the Azores, CE3C - Centre for Ecology, Evolution and Environmental Changes/Azorean Biodiversity Group & CHANGE – Global Change and Sustainability Institute, Rua Capitão João d´Ávila, Pico da Urze, 9700-042, Angra do Heroísmo, Azores, Portugal University of the Azores, CE3C - Centre for Ecology, Evolution and Environmental Changes/Azorean Biodiversity Group & CHANGE – Global Change and Sustainability Institute, Rua Capitão João d´Ávila, Pico da Urze, 9700-042 Angra do Heroísmo, Azores Portugal; 6 IUCN SSC Atlantic Islands Invertebrate Specialist Group, Angra do Heroísmo, Azores, Portugal IUCN SSC Atlantic Islands Invertebrate Specialist Group Angra do Heroísmo, Azores Portugal; 7 Department of Animal Biology, Edaphology and Geology, University of La Laguna, Tenerife, Canary Islands, Spain Department of Animal Biology, Edaphology and Geology, University of La Laguna Tenerife, Canary Islands Spain; 8 University of the Azores, School of Agricultural and Environmental Sciences, Rua Capitão João d´Ávila, Pico da Urze, 9700-042, Angra do Heroísmo, Azores, Portugal University of the Azores, School of Agricultural and Environmental Sciences, Rua Capitão João d´Ávila, Pico da Urze, 9700-042 Angra do Heroísmo, Azores Portugal; 9 Department of Ecology and Taxonomy, Faculty of Biology, National and Kapodistrian University of Athens, Athens, Greece Department of Ecology and Taxonomy, Faculty of Biology, National and Kapodistrian University of Athens Athens Greece; 10 IUCN SSC Monitoring Specialist Group, Angra do Heroísmo, Azores, Portugal IUCN SSC Monitoring Specialist Group Angra do Heroísmo, Azores Portugal

**Keywords:** anthropogenic activities, endemism hotspots, island biogeography, Macaronesia, phenotype, species conservation

## Abstract

**Background:**

Species functional traits provide critical insights into how organisms interact with and respond to their environment. Key characteristics, such as body size, dispersal ability and trophic specialisation influence species' survival, reproduction and adaptability. Island ecosystems, particularly oceanic archipelagos like the Azores, serve as ideal natural laboratories for studying these traits due to their unique biogeographic history and high endemism. Arthropods, as dominant colonisers and ecosystem engineers, exhibit rapid adaptation and trait diversification in these isolated settings. However, island arthropods face escalating threats from habitat loss, climate change and invasive species, which disrupt ecological functions and increase extinction risks. Under the scope of BALA (Biodiversity of Arthropods from the Laurisilva of Azores) project (1999-2021) and SLAM (Long Term Ecological Study of the Impacts of Climate Change in the natural forest of Azores) project (2012-2025), we obtained a comprehensive and standardised dataset of arthropods functional traits currently known to occur in the Azores Archipelago.

**New information:**

We present a standardised functional trait database for Azorean arthropods, building on 25 years of research on 602 species and subspecies across seven classes and 27 orders. The dataset includes endemic, native non-endemic and exotic species, with traits selected for their relevance to disturbance responses (e.g. body size, dispersal, verticality) and ecological interactions (e.g. trophic level, feeding behaviour). By synthesising these data, we aim to support predictive modelling of biodiversity responses to environmental change and provide information for conservation strategies. This resource provides a foundation for global comparisons and advances in trait-based ecology in island systems.

## Introduction

Functional traits are quantifiable attributes expressed at the individual level, encompassing morphological, physiological, biochemical, behavioural, life-history, habitat-related and ecological characteristics, that help us understand how species interact and respond to the environment (Merckx et al. 2018, Wong et al. 2018, Chichorro et al. 2022a, Baeckens et al. 2023, Martins et al. 2023, Schleuning et al. 2023). For instance, the body size of individuals can serve as proxy of the amount of energy it needs to survive, as well as its lifespan, fecundity, clutch size or home range, where larger-bodied species generally require more resources than smaller-bodied ones (Merckx et al. 2018, Wong et al. 2018, Chichorro et al. 2022a, Baeckens et al. 2023, Martins et al. 2023, Schleuning et al. 2023). Additionally, an individual's capacity to avoid inbreeding and to seek out more favourable habitats for survival is linked to its dispersal ability (Kehoe et al. 2020, Chichorro et al. 2022a, Chichorro et al. 2022b). Thus, individuals with greater dispersal capacity may have higher fitness under environmental change than those with limited dispersal (Kehoe et al. 2020, Chichorro et al. 2022a, Chichorro et al. 2022b). Finally, an individual's primary food source and feeding strategy provide insights into its trophic level and preferences; for instance, individuals with a more generalist diet, such as omnivores, may have higher survivability under stress compared to those with a specialised diet, like carnivores (Kehoe et al. 2020, Munstermann et al. 2021, Reuter et al. 2023).

Within the field of functional trait ecology, island ecosystems serve as natural laboratories that can offer unique insights (Whittaker et al. 2017). Due to the way oceanic islands emerge lifeless from volcanic activity (Whittaker et al. 2008, Arjona et al. 2018, Whittaker et al. 2023), only species with specific functional traits are more likely to successfully colonise these environments and establish sustainable populations (Russell and Kueffer 2019). Amongst these organisms, arthropods, the most diverse group of animals, are key colonisers (Russell and Kueffer 2019), playing essential ecosystem roles, such as pollination and nutrient cycling (Noriega et al. 2018, Chapin 2022, Cardoso et al. 2025a). With life histories and generation lengths that can range from a few days to many years (Chapin 2022), many arthropods can rapidly and successfully adapt to new environments (Aria 2022, McCulloch and Waters 2022). As seen in other taxonomic groups (Garcia-Verdugo et al. 2020), this rapid adaptation fosters the evolution of unique functional traits on islands (Whittaker et al. 2014, Russell and Kueffer 2019). As a result, oceanic island arthropods exhibit high levels of endemism, hosting clades of species that cannot be found anywhere else in the world (Russell and Kueffer 2019, Montgomery et al. 2020).

Despite such uniqueness, island arthropod communities face mounting threats from multiple human-induced pressures, particularly land-use change, climate change and species invasions (Terzopoulou et al. 2015, Ferreira et al. 2016, Whittaker et al. 2017, Oyarzabal et al. 2024). Land-use changes reduce and fragment native habitats, while climate change alters temperature and precipitation regimes, narrowing the arthropods’ physiological tolerances (Harvey et al. 2022). At the same time, introduced exotic species may compete with or predate/parasitise on endemic arthropods, often excluding endemics from the habitats they are functionally adapted to (Wong et al. 2020, Harvey et al. 2022). These drivers act simultaneously, often synergistically, to erode the endemic biota and disrupt ecological processes (Uhler et al. 2021, Harvey et al. 2022). Hence, it is essential to understand how these trait-based patterns are shaped by, and respond to, anthropogenic stressors in order to forecast extinction risks, identify resilience mechanisms and design targeted conservation strategies (Oyarzabal et al. 2024, Oyarzabal et al. 2025a, Oyarzabal et al. 2025b). As such, assessing functional diversity offers a powerful framework for capturing both current impacts and future vulnerabilities of arthropod assemblages on oceanic islands.

Several trait databases are available for the Azorean biota, namely for vascular plants (Schaefer et al. 2011), bryophytes (Henriques et al. 2017), spiders (Macías-Hernández et al. 2020), a limited number of other arthropod species (Whittaker et al. 2014, Rigal et al. 2018, Oyarzabal et al. 2024, Wallon et al. 2024) and marine meio-, macro- and megafauna (Campanyà-Llovet et al. 2023, Costa et al. 2023). Yet, most taxa, namely most arthropod orders, are still lacking such data. There is a need to compile and maintain a consistent and standardised database of functional traits for Azorean arthropods, crucial to provide a scientific foundation for testing a wide range of biological and ecological hypotheses. We focused on filling knowledge gaps that will support the development of advanced predictive models, for both present and future scenarios of local and global environmental changes. We curated more than 25 years of arthropod knowledge through an extensive review of the literature (Borges and Wunderlich 2008, Rigal et al. 2018, Macías-Hernández et al. 2020, Costa and Borges 2021, Borges et al. 2022a, Borges et al. 2022b, Borges et al. 2022c, Lhoumeau et al. 2022, Borges et al. 2024, IUCN 2024, Oyarzabal et al. 2024, Pozsgai et al. 2024, Wallon et al. 2024, Oyarzabal et al. 2025a). Amongst the traits included are key life history and ecological attributes of arthropods such as body size, dispersal ability, vertical habitat occupancy and trophic level.

## General description

### Purpose

Here, we aim to provide a comprehensive and standardised dataset of arthropods functional traits currently known to occur in the Azores Archipelago. Within this dataset, we added information we compiled on the origins of each species or rather their means of establishment, clarifying whether they are: i) Azorean endemics, species found naturally only in this Archipelago; ii) native non-endemic, species that, while naturally occurring in the Azores, also inhabit other Macaronesian archipelagos (Madeira, the Canary Islands and Cabo Verde) and/or western Europe; iii) introduced exotics, species known to be cosmopolitan or believed to have reached the Azores, intentionally or unintentionally, by human actions; and iv) indeterminate, species of unknown origin, but more likely to be native non-endemic or exotics (Borges et al. 2006, Borges and Wunderlich 2008, Borges et al. 2022a). Moreover, we focused on traits for which we had a priori expectations regarding their potential responses to increasing habitat disturbance (e.g. body size, dispersal ability, diel activity, habitat presence), traits that play a key role in species interactions (e.g. trophic level, type of food consumption, feeding behaviour) and traits that are broadly comparable across many different terrestrial arthropod taxa (Gossner et al. 2015, Simons et al. 2016, Rigal et al. 2018, Chichorro et al. 2022a, Oyarzabal et al. 2024). All traits are accompanied by a general description of why we hypothesise that they be relevant in future studies (Table 1). By compiling morphological, physiological, biochemical, behavioural, life-history, habitat-related and ecological traits across taxonomic groups and origins, this work provides a foundational resource for analysing biodiversity patterns and understanding species’ responses to environmental change. Moreover, this trait dataset is intended to support ongoing and future ecological studies in the Azores and elsewhere, contributing to major global efforts in the area, such as the Global Repository for Insect Traits (Cardoso et al. 2025b) and the World Spider Trait database (Pekár et al. 2021).

## Sampling methods

### Sampling description

We selected taxa (species and subspecies), based on their known occurrences in both natural and anthropogenic habitats of the Azores and on the availability of, at least, one corresponding trait datum. The taxonomic and functional trait information presented in this dataset originates from a long-term, continuously curated dataset maintained in our laboratory over almost 30 years of research, led and/or supervised by the last author (PAVB). While this dataset integrates information reported across numerous published scientific works, including biodiversity data papers, ecological studies, IUCN Red List assessments and species lists, it cannot be fully compiled from those publications alone. Instead, the published works were themselves informed by this evolving, internally curated dataset, which has been updated, expanded over time and presented here (Borges and Wunderlich 2008, Rigal et al. 2018, Macías-Hernández et al. 2020, Costa and Borges 2021, Borges et al. 2022a, Borges et al. 2022b, Borges et al. 2022c, Lhoumeau et al. 2022, Borges et al. 2024, IUCN 2024, Oyarzabal et al. 2024, Pozsgai et al. 2024, Wallon et al. 2024, Oyarzabal et al. 2025a).

### Quality control

Species and subspecies names were based on the updated checklist of arthropods from the Azores (Borges et al. 2022c).

## Geographic coverage

### Description

**Figure 1.**
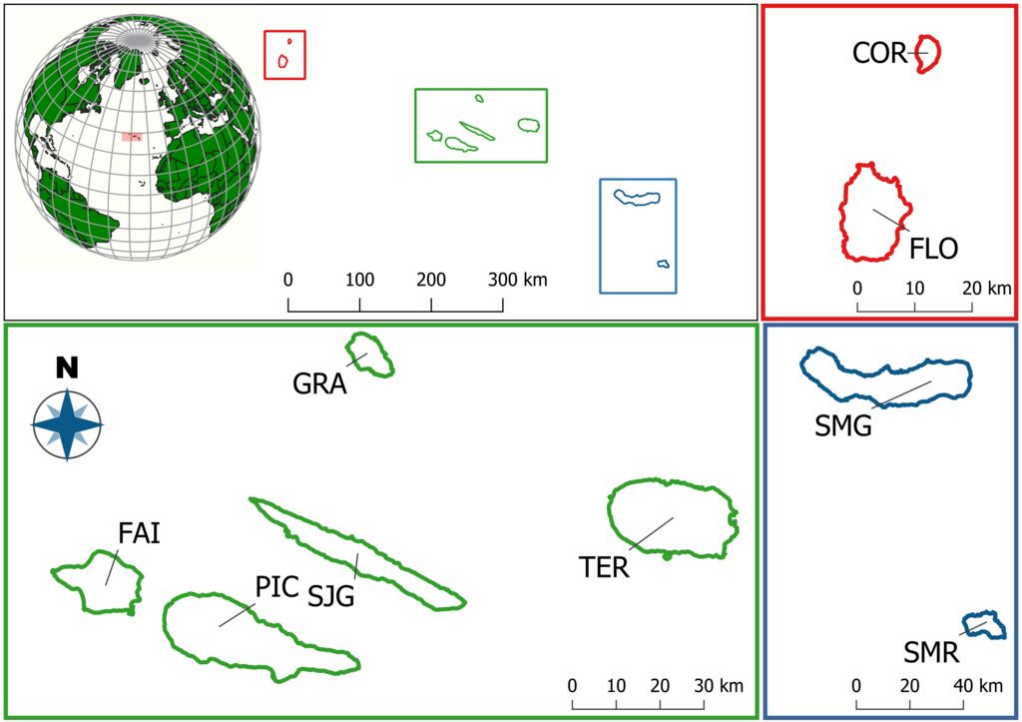
Map of the Azores Archipelago. Top left shows the position of the three island groups: Western group as red for Corvo (COR) and Flores (FLO) islands; Central group as green for Faial (FAI), Pico (PIC), São Jorge (SJG), Graciosa (GRA) and Terceira (TER) islands; and Eastern group as blue for São Miguel (SMG) and Santa Maria (SMR) islands. Adapted from Oyarzabal et al. (2025b).

We conducted this study in the Azores Archipelago, Portugal, which is composed of nine volcanic islands and islets divided into three geographic clusters: the western group (Corvo and Flores), the central group (Faial, Pico, São Jorge, Graciosa and Terceira) and the eastern group (São Miguel and Santa Maria) (Fig. 1). The Azores Archipelago is located in the North Atlantic Ocean, between approximately 37° to 40° N latitude and 24° to 31° W longitude, spanning about 615 km from east to west (Fig. 1). The Archipelago experiences a temperate oceanic climate, characterised by consistently high atmospheric humidity, particularly in the elevated native evergreen laurel forests (Elias et al. 2016, Borges et al. 2022a). Since human colonisation in the 15^th^ century, the Azorean landscape has undergone profound changes, with native habitats increasingly replaced by urban development, exotic forests and agricultural systems (Gaspar et al. 2008, Norder et al. 2020). As a consequence, native forests now account for less than 5% of the total land area, primarily confined to remote or topographically challenging regions that have escaped intensive human disturbance (Gaspar et al. 2008, Triantis et al. 2010, Elias et al. 2016, Lhoumeau et al. 2025).

### Coordinates

36.929555 and 39.721944 Latitude; −31.275000 and -24.781667 Longitude.

## Taxonomic coverage

### Description

The dataset covers 571 species and 31 subspecies of terrestrial arthropods (602 taxa), from seven classes, 27 orders, 172 families and 420 genera (Fig. 2a). We focus preferentially on the endemic taxa with 198 species and 18 subspecies being covered (216 taxa, 78% of known endemic taxa for the Azores; Borges et al. (2022c)). In addition, 115 species and six subspecies are native non-endemic (121 taxa), 197 species and four subspecies are introduced exotics (201 taxa) and 61 species and three subspecies have indeterminate origin (64 taxa). Globally, we covered 24% of Azorean arthropod fauna, which currently totals 2,420 species and subspecies (Borges et al. 2022c). The five most represented orders are: i) Coleoptera (beetles) with 195 species and 16 subspecies (211 taxa); ii) Araneae (spiders) with 119 species; iii) Hemiptera (true bugs) with 60 species and eight subspecies (68 taxa); iv) Lepidoptera (butterflies and moths) with 51 species and one subspecies (52 taxa) and; v) Sarcoptiformes (mites) with 38 species and five subspecies (43 taxa), which cover 81% of all arthropod taxa included here (Fig. 2a). The less represented orders, each with only one species, are Blattodea (cockroaches), Chordeumatida (sausage millipedes), Lithobiomorpha (stone centipedes), Scolopendromorpha (bark centipedes), Strepsiptera (twisted-wing parasites) and Trichoptera (caddisflies) (Fig. 2b). When available, the category given to species according to the IUCN Red List assessments of extinction risk was added to the species information. Taxonomic nomenclature follows the most recent Azorean arthropod checklist by Borges et al. (2022c) and all taxon names are formatted according to Darwin Core standards to ensure interoperability and consistency.

## Traits coverage

The dataset includes a comprehensive combination of 60 traits that describe ecological, morphological, physiological, behavioural, life-history and habitat-related characteristics of the arthropod taxa evaluated in this study (see Table 1). These traits were carefully selected, based on their demonstrated relevance in predictive modelling frameworks aimed at assessing the impacts of human alterations, extinction risk and potential invasiveness on terrestrial arthropod communities (Campomizzi et al. 2008, Rigal et al. 2018, Kehoe et al. 2020, Munstermann et al. 2021, Chichorro et al. 2022a, Chichorro et al. 2022b, Costa et al. 2023, Reuter et al. 2023, Oyarzabal et al. 2024). Trait data were extracted directly from published studies containing measurements or observations from Azorean arthropod populations. Values were used exactly as reported in the original sources, with minimal transformation; unit conversion was applied only when necessary to match our standardised format (e.g. converting centimetres to millimetres), while all other measurements were retained as originally published. Except for Lepidoptera feeding traits, based on larval mouthparts, all traits refer to adult life stages (Rigal et al. 2018). Discrepancies between sources were resolved by harmonising terminology to the controlled vocabulary used in our dataset. For example, species reported to feed on wood parts or soft plant tissues were generalised to the broader category “herbivore”. These steps ensured consistency across traits while preserving the integrity of the original data.

### Data coverage

**Figure 3.**
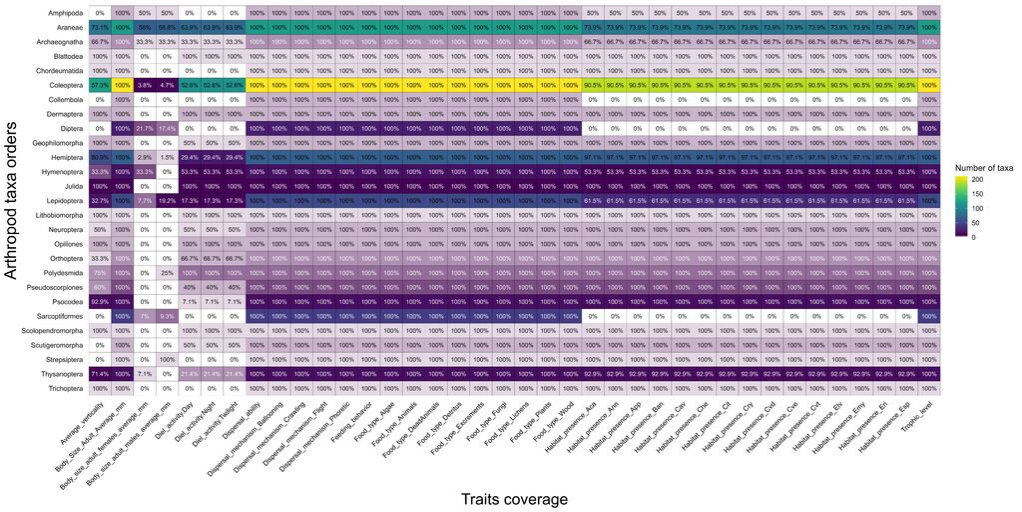
Heatmap of the traits covered by arthropod orders. Colour intensity indicates the number of taxa (species and subspecies) compiled for each trait. The percentage indicates the amount of taxa possessing that trait relative to the total number of taxa recorded for its specific order. Arthropod orders are organised in alphabetical order, from top to bottom. Traits are organised, based on their entries in the main database, from left to right.

From the 602 taxa of terrestrial arthropods compiled, we were able to obtain the full 60 ecological and life-history traits for 50 taxa, 48 species and two subspecies, comprising 15 endemics, eight native non-endemic, 26 introduced exotics and one indeterminate. Within these traits, the average of adult's body size, food type, trophic level, feeding behaviour, dispersal mechanism and dispersal ability were obtained for all 602 species. However, we lacked information on the average body size of adult females for 503 taxa (175 endemic, 108 native non-endemic, 157 introduced exotics and 63 indeterminate), on the average body size of adult males for 499 taxa (169 endemic, 111 native non-endemic, 156 introduced exotics and 63 indeterminate), on all habitat occupancy traits (habitat presence, habitat type and number of habitats) for 151 species (96 endemic, 15 native non-endemic, 29 introduced exotics and 11 indeterminate), on the average verticality for 265 species (129 endemic, 38 native non-endemic, 67 introduced exotics and 31 indeterminate) and on the diel activity for 347 species (184 endemic, 71 native non-endemic, 71 introduced exotics and 21 indeterminate) (Fig. 3).

## Usage licence

### Usage licence

Creative Commons Public Domain Waiver (CC-Zero)

## Data resources

### Data package title

A Comprehensive Trait Dataset for Terrestrial Arthropods of the Azores: Insights for conservation, island ecology and species invasion

### Resource link

Island lab: https://islandlab.uac.pt/software/ver.php?id=53; Zenodo: DOI 10.5281/zenodo.17830286

### Number of data sets

1

### Data set 1.

#### Data set name

Azorean archipelago arthropod life histories and ecological traits.

#### Data format

A file as .csv (Semicolons Separated Values), composed by a simple text.

#### Character set

UTF-8

#### Description

Definition of taxonomical information of the species.

**Data set 1.** 

Column label	Column description
IDcode	An external identifier code, composed of letters and numbers for each species or subspecies presented in alphabetical order. AZO stands for Azores and ART stands for arthropods, followed by counting of species and subspecies in alphabetical order.
speciesCode	An internal identifier code, composed of letters and numbers, for the species or subspecies presented. It follows the species codification used by P.B. during research. MF stands for Morpho-Species, followed by number. When blank, a Morpho-Species number was not designated by P.B.
scientificName	Species or subspecies full scientific name according to the most recent taxonomy and GBIF backbone.
kingdom	The scientific name of the kingdom in which the species or subspecies is classified.
phylum	The scientific name of the phylum in which the species or subspecies is classified.
class	The scientific name of the class in which the species or subspecies is classified.
order	The scientific name of the order in which the species or subspecies is classified.
family	The scientific name of the family in which the species or subspecies is classified.
genus	The scientific name of the genus in which the species or subspecies is classified.
scientificNameAuthorship	Name of the species author, following the International Code of Zoological Nomenclature.
taxonRank	Indicate if the presented taxon is a species or a subspecies.
establishmentMeans	Identify species origin as: i) Endemic, as species only found in the archipelago of Azores; ii) Native, as native non-endemic species that, while naturally occurring in the Azores, also inhabit other Macaronesian archipelagos (Madeira, the Canary Islands and Cabo Verde) and/or western Europe; iii) Introduced exotics, as exotic species that are known to be cosmopolitan or believed to have reached Azores, inadvertently or not, by human actions; and iv) Indeterminate, as species that are unknown in origin, but are more likely to be native non-endemic or introduced exotics.
iucnCategory	Category given to species or subspecies according to the IUCN Red List assessments of extinction risk. It is defined as EX – Extinct, EW – Extinct in the wild, CR – Critically endangered, EN – Endangered, VU – Vulnerable, NT – Near threatened, LC – Least concern and DD – Data deficient. When blank, the species or subspecies were not assessed by the IUCN Red List.

## Additional information

### Potential applications

This functional trait dataset is designed to support predictive modelling of the response of arthropods to global change, enabling both broad-scale assessments across taxa and targeted conservation strategies. The key traits presented here may serve as critical indicators of vulnerability that could guide conservation interventions like habitat restoration. This dataset may also support studies on species interactions, such as predator-prey dynamics or pollination networks, by categorising organisms, based on their functional roles (e.g. herbivores, predators, detritivores) and (micro-)habitat (both presence and verticality). Additionally, comparing traits amongst and across endemic, native non-endemic, indigenous (endemic + native non-endemic) and introduced exotic species helps identify why some exotic species thrive in new environments, while others fail, offering insights into invasion biology. Beyond the Azores Archipelago, this standardised trait dataset might enable cross-regional comparisons, helping scientists to model biodiversity responses to climate change, land-use shifts and other global stressors.

By integrating functional traits into conservation planning, policy-makers can prioritise species or habitats that are functionally irreplaceable or particularly sensitive to disturbance (Pavoine and Ricotta 2023). Overall, this dataset provides a foundation for both theoretical and applied research in ecology, biogeography and ecosystem management.

### Limitations

Although we aimed to make this dataset as comprehensive as possible, it has some key limitations. First, although we included intraspecific variation between female and male body sizes, the values are averaged; hence, variability was inevitably lost. Second, these same averaged trait values were not counted for differences in behaviour, size or habitat use between juveniles and adults (Costa et al. 2023). Third, we did not account for potential intraspecific variation on traits across the habitats and distribution ranges of the species. This standardisation was required due to data availability constraints, as the majority of trait databases and published studies report aggregated values rather than individual measurements. Fourth, for the 'Food type' categories ‘Animals’ and ‘Dead Animals’, the dataset does not distinguish between vertebrates and invertebrates. A finer taxonomic resolution was not possible because many source records did not specify the group, limiting our ability to separate these categories reliably. Furthermore, we acknowledge that most of the species lack information, particularly endemic species (see above the *Data coverage* section). This is due to their rarity in Azorean habitats, the lack of taxonomical specialists to properly identify the species or the lack of further studies with these species. Although this dataset encompasses over 600 species, it may only represent a fraction (about 24%) of the arthropod species that inhabit the Azorean natural and anthropogenic habitats. This gap stems primarily from data deficiencies, as research efforts have historically focused on larger, more charismatic taxa (Cardoso 2012), becoming more consistent only in the past 25 years with the insurgence of multiple projects led by PAVB. Finally, beyond the taxonomic groups and traits covered here, there are still key functionality gaps to be studied, such as physiological thresholds, like temperature and desiccation tolerance and demographic data, like offspring size and reproductive timing. These traits may also be critical to predict responses to global change, but remain scarce for most arthropod species (Logghe et al. 2025).

## Figures and Tables

**Figure 2a.**
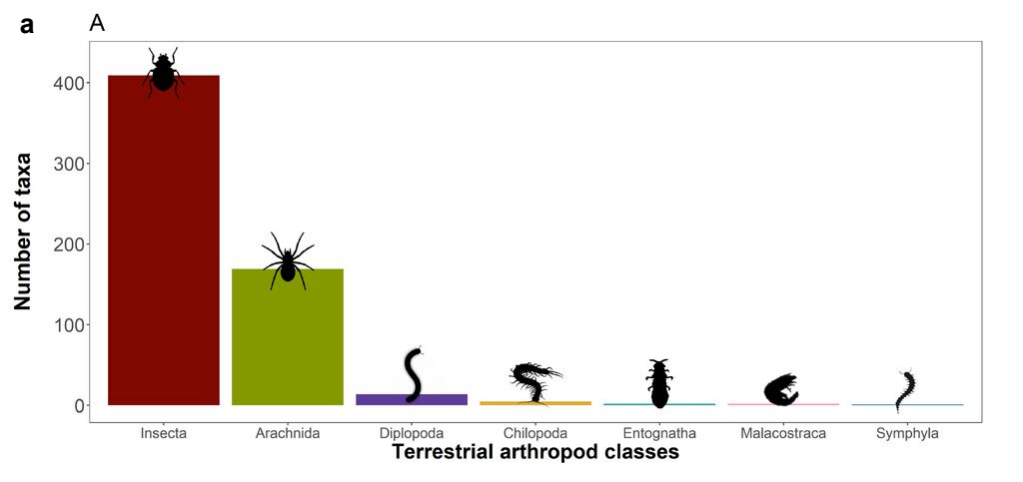


**Figure 2b.**
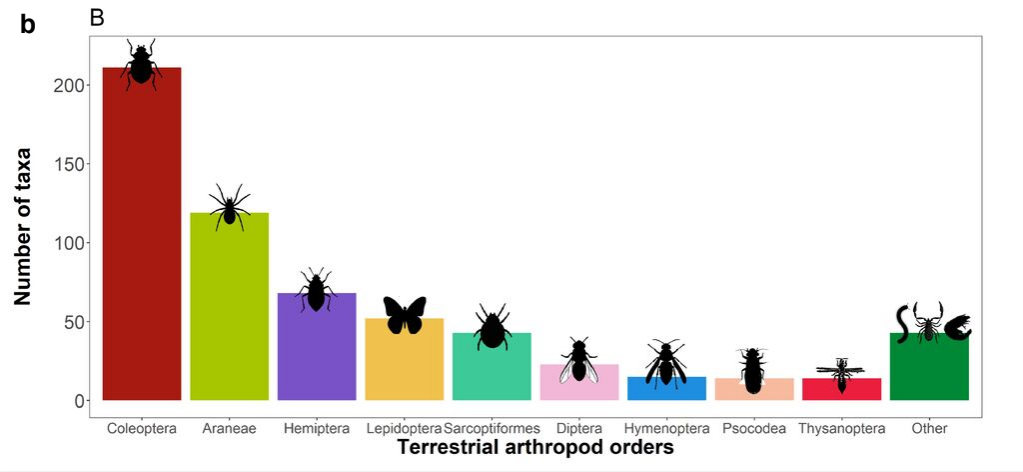


**Table 1.** Definition of the 60 traits for Azorean arthropod species included in the dataset. Some traits are represented as subcategories of broader traits. In such instances, the dataset includes dedicated columns only for the subcategories.

**Trait category**	**Sub-category**	**Type of data**	**Definition and description of the trait**	**General hypothesis**	**References for hypothesis**
Body size adult average		Numerical	Mean adult body length (mm), computed by averaging the body length values of both sexes together (not weighted by sex ratio). Arachnids were measured from the closest point to the chelicera in the prosoma to the end of the abdomen (excluding abdomen appendages). Besides Lepidoptera (butterflies and moths), for which the body length is given by wingspan, all other arthropods (Chilopoda, Diplopoda, Entognatha, Insecta, Malacostraca and Symphyla) body length were given by measuring from the tip of their head to the end of the abdomen (excluding head and abdomen appendages).	Body size mediates species’ responses to environmental change, where larger-bodied species may exhibit lower ecological flexibility than smaller-bodied ones.	Purvis et al. (2000), Chichorro et al. (2022a), Chichorro et al. (2022b), Oyarzabal et al. (2024)
Body size adult females average		Numerical	Mean body length of adult females in millimetres (mm). Arachnids were measured from the closest point to the chelicerae in the prosoma to the end of the abdomen (excluding abdomen appendages). Besides Lepidoptera (butterflies and moths), for which the body length is given by wingspan, all other arthropods (Chilopoda, Diplopoda, Entognatha, Insecta, Malacostraca and Symphyla) body length were given by measuring from the tip of their head to the end of the abdomen (excluding head and abdomen appendages). When blank, the information is not available.	Body size mediates species’ responses to environmental change, where larger-bodied species may exhibit lower ecological flexibility than smaller-bodied ones.	Purvis et al. (2000), Chichorro et al. (2022a), Chichorro et al. (2022b), Oyarzabal et al. (2024)
Body size adult males average		Numerical	Mean body length of adult males in millimetres (mm). Arachnids were measured from the closest point to the chelicerae in the prosoma to the end of the abdomen (excluding abdomen appendages). Besides Lepidoptera (butterflies and moths), for which the body length is given by wingspan, all other arthropods (Chilopoda, Diplopoda, Entognatha, Insecta, Malacostraca and Symphyla) body length were given by measuring from the tip of their head to the end of the abdomen (excluding head and abdomen appendages). When blank, the information is not available.	Body size mediates species’ responses to environmental change, where larger-bodied species may exhibit lower ecological flexibility than smaller-bodied ones.	Purvis et al. (2000), Chichorro et al. (2022a), Chichorro et al. (2022b), Oyarzabal et al. (2024)
Food type		Nominal with multichoice of nine levels as binary values	These are the known food sources for each species, defining if the species feeds on algae, animals, dead animal flesh, detritus, excrement, fungi, lichens, soft plants tissues or plant wood.	Arthropod species with narrow dietary specialisation (e.g. feeding exclusively on one item) are more vulnerable to environmental change and habitat disturbance than generalist feeders, due to their dependence on specific and potentially scarce or habitat-sensitive food resources.	Kehoe et al. (2020), Munstermann et al. (2021), Chichorro et al. (2022a), Chichorro et al. (2022b), Reuter et al. (2023), Oyarzabal et al. (2024)
	Algae		0: the species is not known for feeding on algae; 1: the species is known for feeding on algae.		
	Animals		0: the species is not known for feeding on animals; 1: the species is known for feeding on animals.		
	Dead Animals		0: the species is not known for feeding on dead animals; 1: the species is known for feeding on dead animals.		
	Detritus		0: the species is not known for feeding on detritus; 1: the species is known for feeding on detritus.		
	Excrement		0: the species is not known for feeding on excrement; 1: the species is known for feeding on excrement.		
	Fungi		0: the species is not known for feeding on fungi; 1: the species is known for feeding on fungi.		
	Lichens		0: the species is not known for feeding on lichens; 1: the species is known for feeding on lichens.		
	Plants		0: the species is not known for feeding on plants' soft tissues; 1: the species is known for feeding on plants' soft tissues.		
	Wood		0: the species is not known for feeding on woody parts of plants; 1: the species is known for feeding on woody parts of plants.		
Trophic level		Nominal, with four levels	Expresses the main diet of the species based on the food type known. Defined as: i) Carnivores, only feed on animals, ii) Fungivores, only feed on fungi, iii) herbivores, only feed on algae, lichens, soft plant parts and/or woody plant parts; and iv) omnivores, feed on more than one of the above categories or feed on detritus and/or excrements.	Arthropod species occupying higher or more specialised trophic levels, such as strict carnivores, are more susceptible to environmental change and habitat disturbance than species at lower or more generalist trophic levels.	Kehoe et al. (2020), Munstermann et al. (2021), Chichorro et al. (2022a), Chichorro et al. (2022b), Reuter et al. (2023), Oyarzabal et al. (2024)
Feeding behaviour		Nominal, with three levels	Determines feeding strategies and diet specialisation through mouthparts type. Influencing nutrient cycling and energy flow within ecosystems. Chewing and cutting (CC); piercing and sucking (PS); external digestion and sucking (EDS).	Specialised mouthparts may cause vulnerability to environmental change because specialised mechanisms often limit species to specific food sources.	Gossner et al. (2015), Welti et al. (2020), Wallon et al. (2024)
Average verticality		Numerical	Mean vertical stratum occupancy values, ranging from 0 to 1, represent the proportional distribution of a species across different heights, from the ground level to the highest sampled point in the vegetation. This metric indicates where the species was most frequently observed, whether near the ground or closer to the canopy. When blank, the information is not available.	The vertical stratification of arthropod species may influence their response to environmental change, with ground-dwellers being more vulnerable to general land-use disturbances, while canopy-dwellers may be more protected when inhabiting forest trees.	Rocha-Ortega et al. (2019), Kehoe et al. (2020), Chichorro et al. (2022a), Chichorro et al. (2022b), Costa et al. (2023), Zhang et al. (2023), Oyarzabal et al. (2024)
Diel activity		Nominal with multichoice of three levels as binary values	Refers to the period of the day during which a species is most active. This trait can influence how species interact with their environment, including foraging behaviour, exposure to light, temperature fluctuations and interactions with other organisms.	Compared with strict nocturnal, species with strict diurnal activities may be more susceptible to impact due to a higher exposure to extreme temperatures and increased chance of encounters with humans.	Ockendon et al. (2014), Rigal et al. (2018), Wallon et al. (2024)
	Day		0: the species was not observed or is not known to have a daylight activity pattern; 1: the species was observed or is known to have daylight activity pattern. When blank, the information is not available.		
	Night		0: the species was not observed or is not known to have a nocturnal activity pattern; 1: the species was observed or is known to have nocturnal activity pattern. When blank, the information is not available.		
	Twilight		0: the species was not observed or is not known to have a twilight activity pattern; 1: the species was observed or is known to have a twilight activity pattern. When blank, the information is not available.		
Dispersal mechanism		Nominal with multichoice of four levels as binary values	Dispersal mechanism is determined by the species’ main known behaviour for the dispersion: ballooning (dispersal aided by air currents using silk threads), crawling (active movement on the ground), flying (active movement through the air using self-propulsion, typically with wings), or phoresy (dispersal by attaching to another animal).	Arthropod species employing active or aerial dispersal mechanisms (e.g. ballooning or flying) are more likely to persist and expand their distributions under environmental change than species relying on passive or limited dispersal strategies (e.g. crawling or phoresy).	Rigal et al. (2018), Kehoe et al. (2020), Chichorro et al. (2022a), Chichorro et al. (2022b), Oyarzabal et al. (2024)
	Ballooning		0: the species’ main dispersal method is not through ballooning; 1: the species’ main dispersal method is through ballooning.		
	Crawling		0: the species’ main dispersal method is not through crawling; 1: the species’ main dispersal method is through crawling.		
	Flying		0: the species’ main dispersal method is not through flight; 1: the species’ main dispersal method is through flight.		
	Phoretic		0: the species’ main dispersal method is not through phoresy; 1: the species’ main dispersal method is through phoresy.		
Dispersal ability		Binary	Summarise the species capacity to disperse, considering range and mobility of dispersal. Hence, ballooning and flying species are considered high dispersers (1) and crawling and phoretic are considered poor dispersers (0).	Species with high dispersal ability (i.e. capable of long-range movement via ballooning or flight) may be less impacted by habitat fragmentation and climate change than low-dispersing species (i.e. crawling or phoretic), as they can better relocate and establish in suitable habitats.	Gossner et al. (2015), Rigal et al. (2018), Kehoe et al. (2020), Oyarzabal et al. (2024)
Habitat presence		Nominal with multichoice of thirty-seven levels as binary values	General habitats where the species was sampled at least once or is known to inhabit in the Azores.	Species that are restricted to specific habitat types (e.g. native forests) are more vulnerable to environmental change and habitat degradation than species occurring across a broader range of environments.	Campomizzi et al. (2008), Oyarzabal et al. (2024)
	*Acacia* spp. plants. (Aca)		0: the species was not typically found in samples collected on *Acacia* spp. plants; 1: the species was typically found in samples collected on *Acacia* spp. plants. When blank, the information is not available.		
	*Annona* spp. plants. (Ann)		0: the species was not typically found in samples collected on *Annona* spp. plants; 1: the species was typically found in samples collected on *Annona* spp. plants. When blank, the information is not available.		
	Apple, *Malus* spp., plants. (App)		0: the species was not typically found in samples collected on apple plants; 1: the species was typically found in samples collected on apple plants. When blank, the information is not available.		
	Banana, *Musa* spp., plants. (Ban)		0: the species was not typically found in samples collected on banana plants; 1: the species was typically found in samples collected on banana plants. When blank, the information is not available.		
	Cave habitats. (Cav)		0: the species was not typically found in samples collected in cave habitats; 1: the species was typically found in samples collected in cave habitats. When blank, the information is not available.		
	Cave-dark habitat. (Cvd)		0: the species was not typically found in samples collected in the dark side of cave habitats; 1: the species was typically found in samples collected in the dark side of cave habitats. When blank, the information is not available.		
	Cave-entrance habitat. (Cve)		0: the species was not typically found in samples collected in the cave-entrance habitats; 1: the species was typically found in samples collected in the cave-entrance habitats. When blank, the information is not available.		
	Cave-twilight habitat. (Cvt)		0: the species was not typically found in samples collected in the cave-twilight habitats; 1: the species was typically found in samples collected in the cave-twilight habitats. When blank, the information is not available.		
	Chestnut, *Castanea* spp., plants. (Che)		0: the species was not typically found in samples collected on chestnut plants; 1: the species was typically found in samples collected on chestnut plants. When blank, the information is not available.		
	*Citrus* spp. plants. (Cit)		0: the species was not typically found in samples collected on *Citrus* spp. plants; 1: the species was typically found in samples collected on *Citrus* spp. plants. When blank, the information is not available.		
	*Cryptomeria* spp. plants. (Cry)		0: the species was not typically found in samples collected on *Cryptomeria* spp. plants; 1: the species was typically found in samples collected on *Cryptomeria* spp. plants. When blank, the information is not available.		
	*Erica* spp. plants. (Eri)		0: the species was not typically found in samples collected on *Erica* spp. plants; 1: the species was typically found in samples collected on *Erica* spp. plants. When blank, the information is not available.		
	*Erica* spp. on lava flow habitats. (Elv)		0: the species was not typically found in samples collected on *Erica* spp. that inhabit lava flows; 1: the species was typically found in samples collected on *Erica* spp. that inhabit lava flows. When blank, the information is not available.		
	*Erica* spp.-*Myrica* spp. plants. (Emy)		0: the species was not typically found in samples taken from both *Erica* spp. and *Myrica* spp. plants; 1: the species was typically found in samples taken from both *Erica* spp. and *Myrica* spp. plants. When blank, the information is not available.		
	*Erica* spp. on semi-natural pasture habitats. (Esp)		0: the species was not typically found in samples collected on *Erica* spp. that inhabit semi-natural pastures; 1: the species was typically found in samples collected on *Erica* spp. that inhabit semi-natural pastures. When blank, the information is not available.		
	*Eucalyptus* spp. plants. (Euc)		0: the species was not typically found in samples collected on *Eucalyptus* spp. plants; 1: the species was typically found in samples collected on *Eucalyptus* spp. plants. When blank, the information is not available.		
	Exotic vegetation plants. (Exv)		0: the species was not typically found in samples collected in forests of mixed exotic plants; 1: the species was typically found in samples collected on forests of mixed exotic plants. When blank, the information is not available.		
	*Ficus* spp. plants. (Fic)		0: the species was not typically found in samples collected on *Ficus* spp. plants; 1: the species was typically found in samples collected on *Ficus* spp. plants. When blank, the information is not available.		
	Field margin habitat. (Fie)		0: the species was not typically found in samples obtained at the edge of pasture habitats; 1: the species was typically found in samples obtained at the edge of pasture habitats. When blank, the information is not available.		
	Intensive pasture habitats. (Int)		0: the species was not typically found in samples collected in intensive pasture habitats; 1: the species was typically found in samples collected in intensive pasture habitats. When blank, the information is not available.		
	Lava flow (Lav) habitats.		0: the species was not typically found in samples collected in geologically recent lava flows; 1: the species was typically found in samples collected in geologically recent lava flows. When blank, the information is not available.		
	Mixed habitat. (Mix)		0: the species was not typically found in samples collected in habitats with a mixed composition of endemic and exotic plants; 1: the species was typically found in samples collected in habitats with a mixed composition of endemic and exotic plants. When blank, the information is not available.		
	Native forest habitat. (Nat)		0: the species was not typically found in samples collected in the native Azorean Forest; 1: the species was typically found in samples collected in the native Azorean Forest. When blank, the information is not available.		
	Natural pasture habitat. (Npa)		0: the species was not typically found in samples collected in the natural Azorean pastures; 1: the species was typically found in samples collected in the natural Azorean pastures. When blank, the information is not available.		
	Olive, *Olea* spp., plants. (Oli)		0: the species was not typically found in samples collected on olive plants; 1: the species was typically found in samples collected on olive plants. When blank, the information is not available.		
	Peach, *Prunus* spp., plants. (Pea)		0: the species was not typically found in samples collected on peach plants; 1: the species was typically found in samples collected on peach plants. When blank, the information is not available.		
	Peat bog habitat. (Pbo)		0: the species was not typically found in samples collected in peat bogs habitat; 1: the species was typically found in samples collected in peat bogs habitat. When blank, the information is not available.		
	*Pinus* spp. plants. (Pin)		0: the species was not typically found in samples collected on *Pinus* spp. plants; 1: the species was typically found in samples collected on *Pinus* spp. plants. When blank, the information is not available.		
	*Pittosporum* spp. plants. (Pit)		0: the species was not typically found in samples collected on *Pittosporum* spp. plants; 1: the species was typically found in samples collected on *Pittosporum* spp. plants. When blank, the information is not available.		
	*Pittosporum* and *Myrica* spp. plants. (Pmy)		0: the species was not typically found in samples collected on habitats with a mixed composition of *Pittosporum* and *Myrica* spp. plants; 1: the species was typically found in samples collected on habitats with a mixed composition of *Pittosporum* and *Myrica* spp. plants. When blank, the information is not available.		
	*Protea* spp. plants. (Pro)		0: the species was not typically found in samples collected on *Protea* spp. plants; 1: the species was typically found in samples collected on *Protea* spp. plants. When blank, the information is not available.		
	Semi-natural pasture habitats. (Snp)		0: the species was not typically found in samples collected in semi-natural pasture habitats; 1: the species was typically found in samples collected in semi-natural pasture habitats. When blank, the information is not available.		
	Urban Area habitat. (Urb)		0: the species was not typically found in samples collected in urban areas; 1: the species was typically found in samples collected in urban areas. When blank, the information is not available.		
	Vine, *Vitis* spp. plants. (Vin)		0: the species was not typically found in samples obtained in habitats with a mixed composition of vine plants; 1: the species was not typically found in samples obtained in habitats with a mixed composition of vine plants. When blank, the information is not available.		
Habitat type		Binary	It indicates whether the habitats where the species were sampled are of natural or human origin.	Species predominantly found in human-modified habitats are likely more resilient to anthropogenic pressures than species confined to natural habitats.	Campomizzi et al. (2008), Oyarzabal et al. (2024)
	Anthropogenic habitat.		0: Defines if the species was not sampled in an anthropogenic environment, Aca, Ann, App, Ban, Che, Cit, Cry, Euc, Exv, Fic, Fie, Int, Mix, Oli, Pea, Pin, Pit, Pmy, Pro, Snp, Urb, Vin; 1: Defines if the species was sampled in an anthropogenic environment, Aca, Ann, App, Ban, Che, Cit, Cry, Euc, Exv, Fic, Fie, Int, Mix, Oli, Pea, Pin, Pit, Pmy, Pro, Snp, Urb, Vin. When blank, the information is not available.		
	Natural habitat.		0: Define if the species was not sampled in a natural environment, Cav, Cvd, Cve, Cvt, Eri, Elv, Esp, Emy, Lav, Nat, Npa, Pbo; 1: Define if the species was sampled in a natural environment, Cav, Cvd, Cve, Cvt, Eri, Elv, Esp, Emy, Lav, Nat, Npa, Pbo. When blank, the information is not available.		
Number of habitats present		Numerical	Total number of habitat types in which the species has been sampled or is known to inhabit, calculated as the sum of the preceding "Habitat presence" trait. A blank indicates that no information is available.	Species that may occur in a greater number of habitat types show higher ecological plasticity and resilience, making them less susceptible to extinction under global change scenarios compared to habitat specialists with narrow distributions.	Campomizzi et al. (2008)
